# Comparative Efficacy of Videolaryngoscopy and Direct Laryngoscopy in Patients Living With Obesity: A Meta-Analysis

**DOI:** 10.7759/cureus.76558

**Published:** 2024-12-29

**Authors:** Hannan Chaudery, Harira Hameed, Zaina Sharif, Sheko Asinger, Andrew McKechnie

**Affiliations:** 1 Anaesthesia, King’s College Hospital NHS Foundation Trust, London, GBR; 2 Medicine, Multan Medical and Dental College, Multan, PAK; 3 Medicine, Croydon Health Services NHS Trust, London, GBR; 4 Emergency Medicine, Croydon Health Services NHS Trust, London, GBR; 5 Anaesthesia, Lewisham and Greenwich NHS Trust, London, GBR

**Keywords:** direct laryngoscopy, glidescope, laryngoscopy, meta-analysis, obesity, videolaryngoscopy

## Abstract

Intubation in patients living with obesity poses unique challenges due to altered airway anatomy and reduced physiological reserve, increasing the risk of complications. In synthesizing evidence from multiple trials, our meta-analysis suggests that videolaryngoscopy may provide a higher likelihood of achieving successful intubation on the first attempt compared to direct laryngoscopy while not substantially increasing the procedure time. Videolaryngoscopy was associated with a significant increase in first-pass intubation success compared to direct laryngoscopy, with a pooled risk ratio (RR) of 0.42 (95% CI 0.22 - 0.78, p = 0.0064). There was no significant difference in time to intubation between the two techniques (standardised mean differences (SMD) 0.13, 95% CI -0.26 to 0.52, p = 0.51), a result approached with low certainty due to the high heterogeneity among studies. Although the underlying studies varied in their methods and patient populations, these findings support the consideration of videolaryngoscopy as a potentially more reliable and safer technique for airway management in patients with obesity.

## Introduction and background

Airway management presents unique challenges in patients living with obesity. This patient population presents a higher risk of difficult and failed intubations, both significant contributors to anaesthetic-related morbidity and mortality [1]. This increased risk is due to anatomical and physiological alterations associated with obesity, including reduced oxygen reserve and limited time for airway manipulation before desaturation. The increasing prevalence of obesity worldwide has made this an issue of growing importance in clinical practice.

Direct laryngoscopy (DL) has been the cornerstone of tracheal intubation for decades. However, the advent of videolaryngoscopy (VL) introduced a technology aimed at improving glottic visualisation, potentially enhancing the success rates of first-pass intubation and reducing complications. While VL has been widely adopted in clinical settings, its comparative efficacy against DL, especially in patients living with obesity, remains a subject of debate.

Recent years have seen a proliferation of studies comparing VL and DL in various patient populations. However, findings have been heterogeneous and often underpowered, particularly in the context of obesity [2]. This inconsistency makes it challenging to draw definitive conclusions about the superior technique for ensuring first-pass intubation success in this demographic. Moreover, the impact of these intubation techniques on the time to intubation in emergency airway management, a potentially impactful factor, given shorter apnoea times in this patient population, has not been conclusively determined. 

Two meta-analyses with similar clinical questions have been presented in the past in the literature, but the limited number of articles included and/or the total sample sizes have dampened the generalisability of results. Most recently, a Cochrane review considered the wider question of VL versus DL in all patients but did not find enough data to conduct a subgroup analysis of patients living with obesity [2]. 

Given the clinical importance of optimising airway management in patients living with obesity and the existing gaps in the literature, there is a pressing need for a comprehensive meta-analysis. By systematically reviewing and synthesising available evidence, this study aims to provide the largest and most comprehensive comparison of both the efficacy of VL and DL in achieving first-pass intubation success and evaluate any difference in time to intubation. Such insights are crucial for guiding clinical practice and improving patient outcomes in this high-risk population.

## Review

Methods

A systematic search was conducted across PubMed, Excerpta Medica database (Embase), and Google Scholar databases to identify randomised controlled trials (RCTs) comparing the efficacy of videolaryngoscopy (VL) and direct laryngoscopy (DL) in patients living with obesity. The search strategy was designed to include a broad range of terms related to "videolaryngoscopy," "direct laryngoscopy," "obesity," and "intubation." No language or date restrictions were applied to maximise the inclusivity of relevant studies. The search was last updated on 24/02/24, ensuring a comprehensive review of the literature up to that point. Where results were published without broken-down data, the authors were contacted directly to receive this. The meta-analysis was prospectively registered in the International Prospective Register of Systematic Reviews (PROSPERO) database (registration number CRD42024517529).

Eligibility Criteria

Included studies were RCTs that directly compared VL and DL in obese adults requiring tracheal intubation. Studies were selected based on the following criteria: comparison of VL versus DL, reported outcomes including first-pass intubation success and/or time to intubation, and inclusion of patients living with obesity, defined by a body mass index (BMI) ≥30 kg/m². Exclusion criteria encompassed non-randomised studies, paediatric populations, and studies not reporting the outcomes of interest.

Outcomes

The primary outcome in this analysis was ‘first pass intubation success’ (FPS), meaning successful intubation of the trachea on the first attempt at laryngoscopy. The secondary outcome was time to intubation (TTI), defined as the time taken for the operator to successfully intubate the trachea.

Data Extraction

Two independent reviewers extracted data using a standardised data extraction form. Discrepancies were resolved through discussion or consultation with a third reviewer. Extracted data included study characteristics (authors, year of publication, study design), participant demographics (sample size, mean BMI), intervention details (type of VL and DL devices used), and outcomes (first-pass intubation success, time to intubation). 

Quality Assessment

The risk of bias in included studies was assessed using the Cochrane collaboration’s tool for assessing the risk of bias in randomised trials (risk of bias 2). This evaluation focused on domains such as random sequence generation, allocation concealment, blinding of participants and personnel, blinding of outcome assessment, incomplete outcome data, selective reporting, and other biases, namely the experience of the intubator. 

Statistical Analysis

A random-effects meta-analysis was performed using the restricted maximum-likelihood estimation method to calculate pooled risk ratios (RRs) and standardised mean differences (SMDs). Risk ratios (RR) for binary outcomes and SMD for continuous outcomes were calculated with 95% confidence intervals (CIs). Heterogeneity among studies was quantified using the I² statistic, with values >50% indicating substantial heterogeneity. Sensitivity analyses were conducted to explore the influence of individual studies on the overall results. All analyses were performed using R version 4.3.2 (R Core Team 2021) (R Foundation for Statistical Computing, Vienna, Austria, https://www.R-project.org/) and the metafor package [3]. The methodology and reporting adhered to Preferred Reporting Items for Systematic Reviews and Meta-Analyses (PRISMA) guidelines.

Results

**Figure 1:**
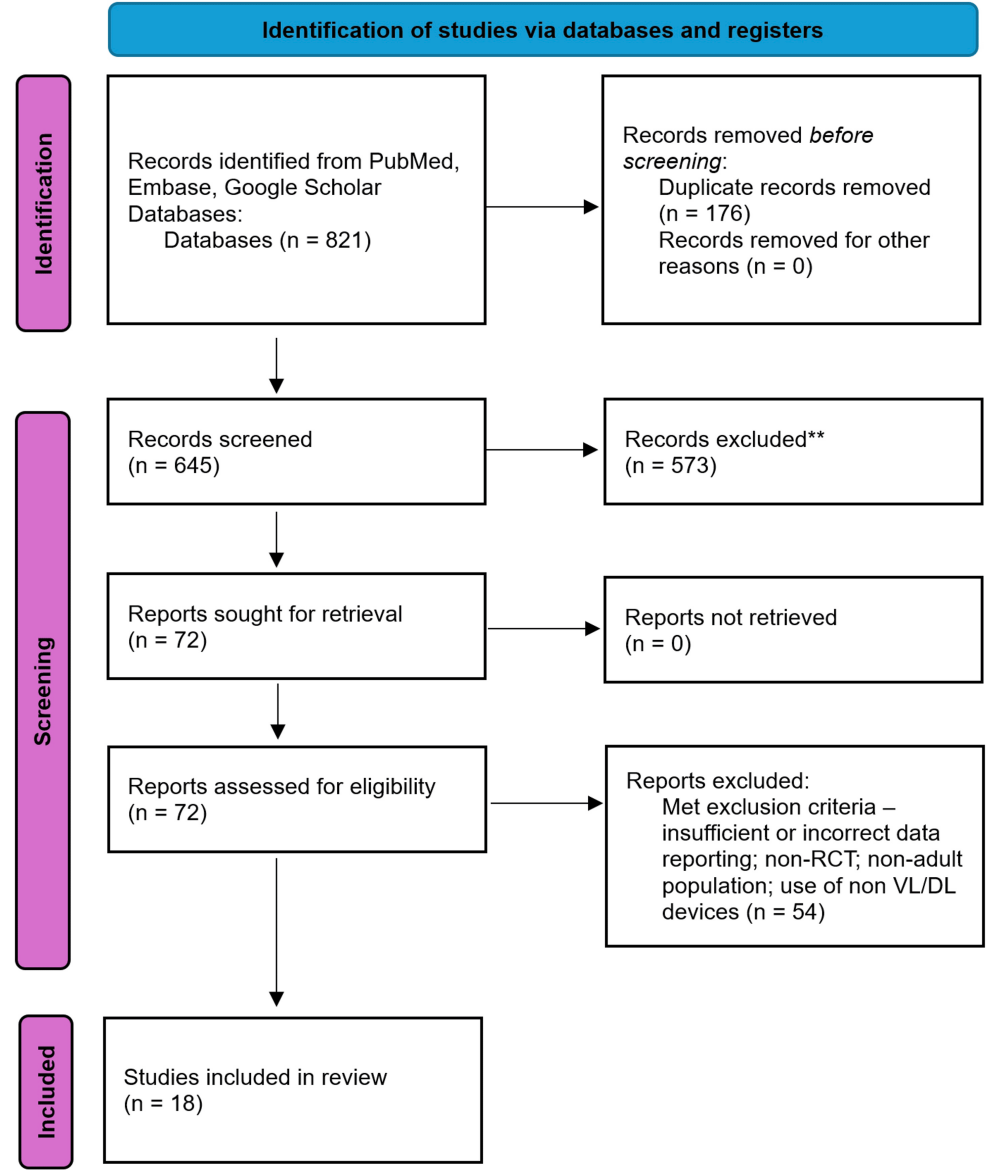
PRISMA flow diagram illustrating screening and study inclusion PRISMA: Preferred Reporting Items for Systematic Reviews and Meta-Analyses; VL: videolaryngoscopy; DL: direct laryngoscopy; RCT: randomised controlled trials [4]

The systematic search across PubMed, Embase, and Google Scholar yielded a total of 821 records. After removing duplicates, 645 titles and abstracts were screened for eligibility, leading to the full-text review of 72 studies. Of these, 18 randomised controlled trials met the inclusion criteria and were included in the meta-analysis. The study selection process is summarised in a PRISMA flow diagram (Figure 1), illustrating the screening and inclusion phases.

The 18 included studies encompassed a total of 1594 patients living with obesity, defined by a BMI ≥30 kg/m² (Table 1). The studies varied in geographical location and included data from North America, Europe, Asia, and the remainder distributed across other regions. The VL devices used across studies included both Macintosh-style and hyper-angulated style blades, while DL was performed with standard laryngoscopes [5-22].

**Table 1:** The study design, sample size, patient characteristics and interventions (method of intubation) of studies included in the meta-analysis BMI: body mass index; ASA: American Society of Anesthesiologists; DL: direct laryngoscopy; VL: videolaryngoscopy [5-22]

Study	Study Design	Sample Size	Patient Characteristics	Intervention
Abdallah et al., 2011 [5]	Randomized Controlled Trial	105	BMI is 30-50 kg/m² and undergoing elective surgery	Pentax AWS (VL) vs. Macintosh (DL)
Akbarzadeh et al., 2017 [6]	Randomized Controlled Trial	102	ASA class I or II and BMI of ≥ 30 kg/m²	GlideScope (VL) vs. Macintosh (DL) vs. McCoy (DL)
Ander et al., 2017 [7]	Randomized Controlled Trial	80	BMI ≥ 35 kg/m²	Storz C-MAC (VL) vs. Macintosh (DL)
Andersen et al., 2011 [8]	Randomized Controlled Trial	100	BMI ≥ 35 kg/m² and undergoing bariatric Surgery	GlideScope (VL) vs. Macintosh (DL)
Çakir et al., 2020 [9]	Randomized Controlled Trial	62	ASA class II and BMI ≥ 35 kg/m²	McGrath (VL) vs. Macintosh (DL)
Castillo-Monzón et al., 2017 [10]	Randomized Controlled Trial	46	ASA class III and BMI >40 kg/m² and age ≥ 18	Airtraq laryngoscope (VL) vs. Macintosh (DL)
Korkusuz et al., 2023 [11]	Randomized Controlled Trial	90	BMI 30+ kg/m² and age ≥ 18	Macintosh (DL) vs. Macintosh + stylet (DL+S) vs. McGrath (VL) vs. McGrath + Stylet (VL+S)
Malik et al., 2009 [12]	Randomized Controlled Trial	75	ASA class I - III and age ≥ 16. Not specific for obesity but mean BMI in all 3 groups was ≥ 30 kg/m²	Pentax AWS (VL) vs. Glidescope (VL) vs. Macintosh (DL)
Marrel et al., 2007 [13]	Randomized Controlled Trial	80	BMI ≥ 40 kg/m²	X-Lite (VL) vs. Macintosh (DL)
Nandakumar et al., 2018 [14]	Randomized Controlled Trial	45	BMI ≥ 40 kg/m²	GlideScope (VL) vs. Macintosh (DL) vs. McCoy (DL)
Ndoko et al., 2008 [15]	Randomized Controlled Trial	106	BMI ≥ 35 kg/m² and undergoing gynae or bariatric surgery	Airtraq laryngoscope (VL) vs. Macintosh (DL)
Postaci et al., 2015 [16]	Randomized Controlled Trial	84	BMI ≥ 30 kg/m² and female sex	McGrath (VL) vs. Macintosh (DL)
Ranieri et al., 2012 [17]	Randomized Controlled Trial	132	BMI ≥ 30 kg/m² and undergoing bariatric surgery	Airtraq laryngoscope (VL) vs. Macintosh (DL)
Rovsing et al., 2010 [18]	Randomized Controlled Trial	100	BMI ≥ 30 kg/m² and undergoing bariatric surgery	GlideScope (VL) vs. Macintosh (DL)
Ruetzler et al., 2020 [19]	Randomized Controlled Trial	130	BMI ≥ 40 kg/m²	McGrath (VL) vs. Macintosh (DL)
Wasinwong et al., 2017 [20]	Randomized Controlled Trial	46	BMI ≥ 28 kg/m² and undergoing elective surgery	GlideScope (VL) vs. MacIntosh (DL)
Yousef et al., 2012 [21]	Randomized Controlled Trial	90	BMI ≥ 35 kg/m² and undergoing gynae and bariatric surgery	GlideScope (VL) vs. LMA Ctrach (VL) vs. MacIntosh (DL)
Yumul et al., 2016 [22]	Randomized Controlled Trial	121	BMI ≥ 30 kg/m² and age ≥ 18 and undergoing elective bariatric surgery	Video-Mac (VL) vs. GlideScope (VL) vs. McGrath (VL) vs. Macintosh (DL)

**Figure 2:**
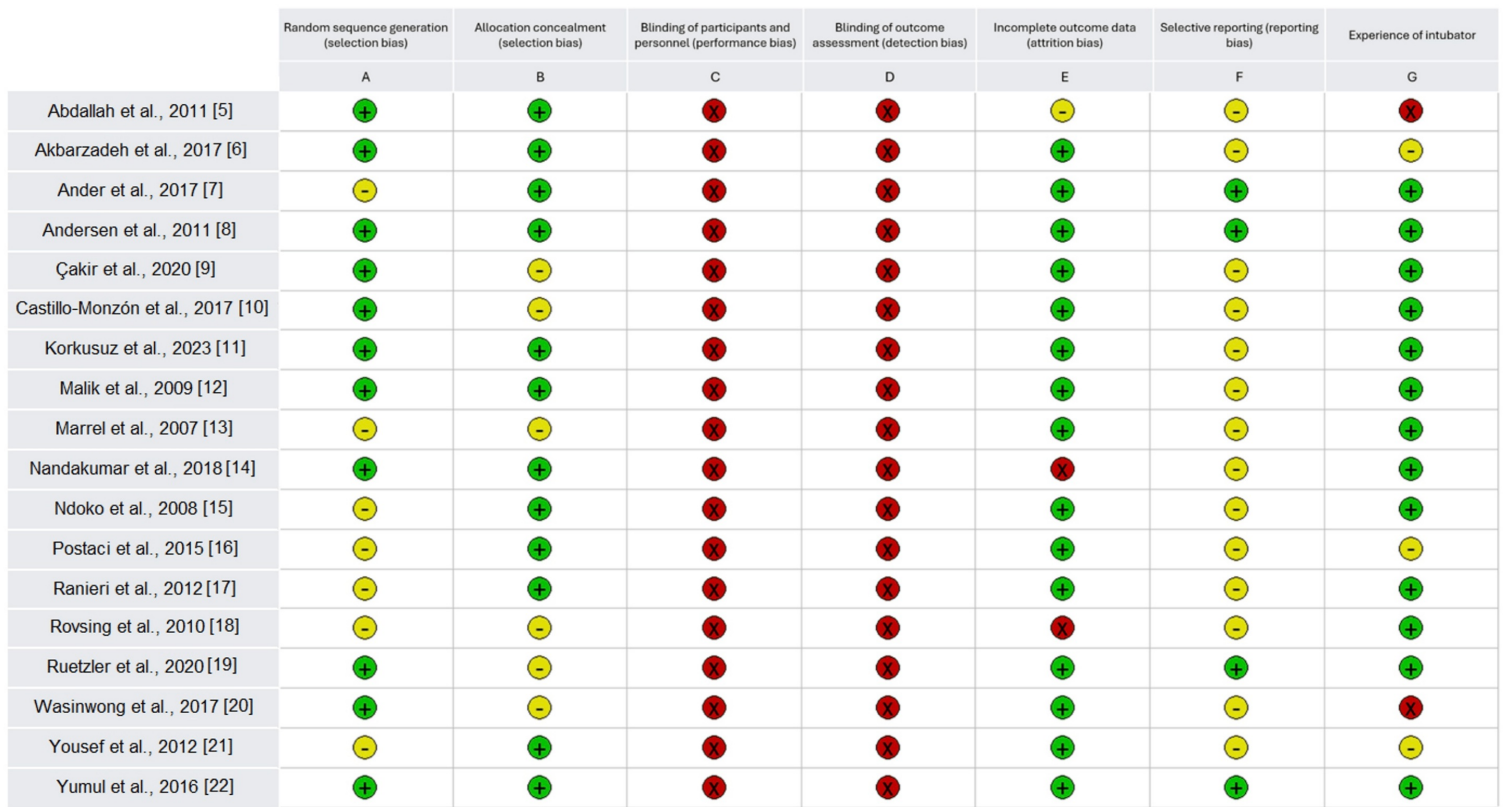
Cochrane risk of bias 2 (ROB2) analysis of included articles Key: (x) indicates risk of bias; (+) indicates no risk of bias; (-) indicates unequivocal risk of bias. [5-22]

Due to the nature of the studies involved, all 18 were at risk of performance and detection bias due to lack of blinding of participants and personnel, as well as blinding of outcome assessment. Most included papers had low to moderate risk of bias in selection and allocation methods, often electing to use appropriate random sequence generation techniques, as illustrated in Figure 2. Two out of 18 studies did not declare the experience of intubators used, three studies included intubators of mixed experience, and the remaining 13 declaring experienced intubators in both device arms. 

First-Pass Intubation Success

**Figure 3:**
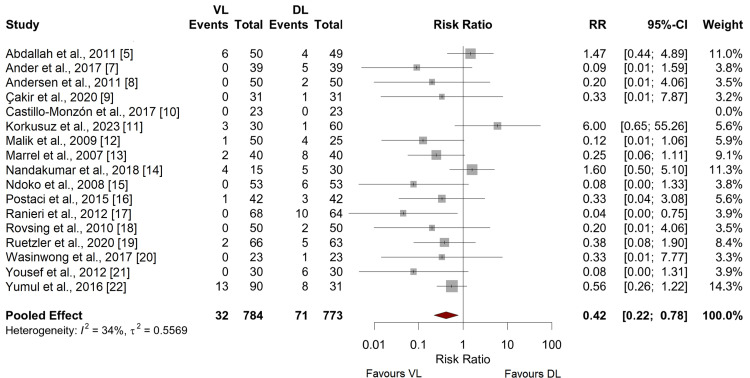
Meta-analysis of first pass intubation success rate [5,7-22]

Videolaryngoscopy was associated with a significantly higher first-pass intubation success rate compared to direct laryngoscopy with a pooled risk ratio (RR) of 0.42 (95% confidence interval (CI) 0.22 - 0.78, p = 0.0064), as illustrated in Figure 3. Moderate certainty was attributed to this outcome, based on the overall risk of bias and consistency across studies. 

Time to Intubation

**Figure 4:**
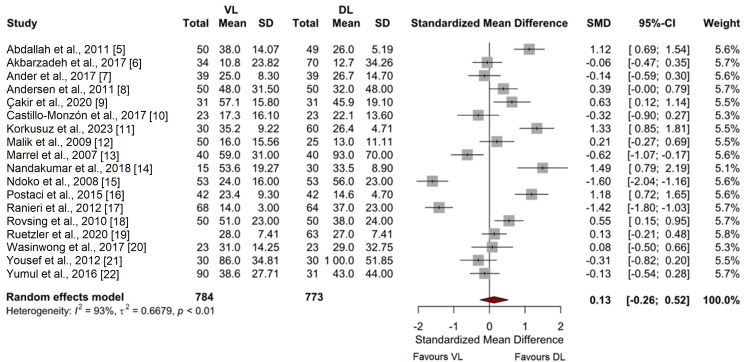
Meta-analysis of time to intubation [5-22]

No significant difference was observed in the time to intubation between VL and DL with a standardized mean difference (SMD) of 0.13 (95% CI -0.26 to 0.52, p = 0.51), as illustrated in Figure 4. This result was approached with low certainty due to high heterogeneity among the included studies (I² = 93%).

Heterogeneity and Sensitivity Analyses

Substantial heterogeneity was observed in the analysis of time to intubation (I² = 93%), which was not significantly reduced in sensitivity analyses excluding studies with high risk of bias. The heterogeneity in first-pass intubation success was moderate (I² = 34%), and sensitivity analyses did not significantly alter the pooled effect estimate.

Discussion

Our meta-analysis reveals that videolaryngoscopy (VL) significantly enhances first-pass intubation success in patients living with obesity compared to direct laryngoscopy (DL), with a pooled risk ratio (RR) of 0.42. This finding underscores the efficacy of VL in navigating the anatomical challenges posed by obesity, aligning with the hypothesis that improved glottic visualisation translates to higher intubation success rates. This result can be partially attributed to factors that make intubation more difficult because of anatomical features in obesity, such as short chin-to-sternum distance, augmented tongue volume, or fixed neck flexion [15]. 

The superiority of VL in achieving first-pass success corroborates findings from previous studies in mixed populations but is particularly pronounced in the obese demographic, where airway management difficulties are exacerbated. A recent Cochrane analysis by Hansel et al. demonstrates similar results in the wider patient population, although analysis of patients living with obesity was not specifically conducted as a subgroup analysis [2]. Secondly, no previous meta-analysis has considered the importance of “first pass success” specifically, choosing to consider intubation success overall as an outcome measure. In this complex and high-risk patient population, we consider first-pass success a better outcome measure due to the added risks of desaturation and hypoxia, as well as further increased airway difficulty with multiple intubation attempts.

Our analysis did not demonstrate a significant difference in time to intubation between VL and DL amongst this population group. This suggests that while VL may facilitate a more successful initial attempt, time to intubation may be influenced by several other factors not mitigated by improved visualisation alone. The notable heterogeneity observed in the analysis of time to intubation likely reflects the diverse methodologies and settings of the included studies, suggesting that factors beyond the choice of laryngoscopy technique play a crucial role in determining the efficiency of intubation.

Time to intubation between VL and DL may be impacted by several confounding factors, such as differences in a patient’s position at the time of intubation. Studies have found that VL was better than DL for patients intubated in the dorsal decubitus position relative to the ‘sniffling’ position [15,23]. Similarly, in the study by Ndoko et al., faster intubation times for VL than DL was found; reference was made to airway position in potentially impairing conditions for tracheal intubation with DL but favouring those for VL (Airtraq) [15]. This suggests that in the studies reviewed, the patient position may have influenced the time to intubation between video laryngoscopy and direct laryngoscopy groups. Unfortunately, not all studies specified patient's position at the time of intubation, and therefore it is difficult to assess the degree to which this may have confounded results in this systematic review. 

User experience may also account for heterogeneity in time to intubation between VL and DL. The seniority of the intubator and length of prior experience with VL devices before attempting difficult intubation differ between studies and were not always quantified. The minimum recommended experience with VL devices prior to attempting VL in difficult airways was not always specified and differs between devices. For example, the Airtaq (VL) recommends a minimum of two to four uses [24]. But even when anaesthetists had used this device on (up to) 15 previous occasions, the time to intubation was longer than with a conventional DL, which anaesthetists generally have significantly more experience with [17]. In contrast, Marrel et al. performed their study with a single senior intubator for both the DL and VL groups with at least six months of experience in VL, which would positively impact the results of the VL group [13]. Inexperience of an intubator with the type of videolaryngoscopy studied may lead to a greater time to intubation, and this should be accounted for when reviewing the existing literature. 

The difficulty of intubation may have also impacted differences in time to intubation between videolaryngoscopy and direct laryngoscopy. Abdallah et al. highlights a shorter time to intubation with direct laryngoscopy in instances where the patients’ heads were not restrained in a collar or subjected to inline stabilization [5]. These factors can both increase the difficulty of intubation by direct laryngoscopy and therefore shorter intubation time may be related to ease of intubation. Similarly, this is supported by studies where time to intubation was longer with videolaryngoscopy than direct laryngoscopy when simulated laryngoscopy scenarios were ‘easy,’ but time to intubation was shorter for VL relative to DL in simulated laryngoscopy scenarios classified as ‘difficult’ [25,26]. The difficulty of intubating an airway was not always equal between the different groups studied. Wasingwong et al. acknowledge that the majority of obese patients in the DL group did not have a difficult airway, unlike the patients in the VL group [20]. A standardised approach was not adopted to measure the ease of intubation in terms of patient positioning, manipulation manoeuvres required, or difficulty of the airway itself. These findings suggest ease of intubation may act as a confounding variable when comparing time to intubation between VL and DL groups, particularly when patient characteristics are not measured, let alone equalised, between the two study groups.

Another possible contributor towards the heterogeneity of time to intubation observed in this study was the varying definitions and methodology used to time the speed of intubation. Likewise, differences in patient comorbidities and specific device characteristics, with differing types of videolaryngoscopy devices used in each study, may also contribute to differences in time to intubation. 

There are several other limitations to this systematic review. The inclusion of studies with varying definitions of obesity, differences in VL and DL devices used, and the relatively small total population size may affect the generalisability of our findings. Furthermore, the moderate certainty of evidence for first-pass success and low certainty for time to intubation necessitate further high-quality research to refine these estimates. Given the ever-increasing average BMI in populations, particularly in Western countries, further studies on BMI ≥35 and BMI ≥40 patients would be particularly useful. With larger datasets, further breakdown of the type of videolaryngoscopy device between Macintosh-style and hyper-angulated devices may inform practice as the technology becomes more ubiquitous.

Despite these limitations, our findings have significant implications for clinical practice. They support the preferential use of VL in patients living with obesity to enhance first-pass intubation success, potentially reducing the risk of hypoxia and other complications associated with multiple intubation attempts. However, the choice of intubation technique should also consider the operator's experience and the specific circumstances of each case.

## Conclusions

This meta-analysis indicates that videolaryngoscopy likely offers a safer approach for airway management in patients living with obesity by improving first-pass intubation success rates compared to direct laryngoscopy. While the time to intubation did not significantly differ between the two techniques, the overall benefit of VL in this high-risk population is evident. These findings advocate for the integration of VL into airway management protocols for these patients, alongside the development of comprehensive training programs to maximise its efficacy. Further research is warranted to explore the nuances of VL and DL performance across diverse clinical scenarios and to establish standardised guidelines that optimise patient outcomes in bariatric populations.
